# Association between cancer‐specific adverse event triggers and mortality: A validation study

**DOI:** 10.1002/cam4.3033

**Published:** 2020-04-13

**Authors:** Saul N. Weingart, Jason Nelson, Benjamin Koethe, Omar Yaghi, Stephan Dunning, Albert Feldman, David Kent, Allison Lipitz‐Snyderman

**Affiliations:** ^1^ Tufts Medical Center Boston MA USA; ^2^ Department of Medicine Tufts University School of Medicine Boston MA USA; ^3^ OptumLabs Cambridge MA USA; ^4^ Predictive Analytics and Comparative Effectiveness Center Tufts University School of Medicine Boston MA USA; ^5^ Memorial Sloan Kettering Cancer Center New York NY USA

**Keywords:** adverse events, epidemiology, oncology, quality of care, trigger tool

## Abstract

**Background:**

As there are few validated measures of patient safety in clinical oncology, creating an efficient measurement instrument would create significant value. Accordingly, we sought to assess the validity of a novel patient safety measure by examining the association of oncology‐specific triggers and mortality using administrative claims data.

**Methods:**

We examined a retrospective cohort of 322 887 adult cancer patients enrolled in commercial or Medicare Advantage products for one year after an initial diagnosis of breast, colorectal, lung, or prostate cancer in 2008‐2014. We used diagnosis and procedure codes to calculate the prevalence of 16 cancer‐specific "triggers"–events that signify a potential adverse event. We compared one‐year mortality rates among patients with and without triggers by cancer type and metastatic status using logistic regression models.

**Results:**

Trigger events affected 19% of patients and were most common among patients with metastatic colorectal (41%) and lung (50%) cancers. There was increased one‐year mortality among patients with triggers compared to patients without triggers across all cancer types in unadjusted and multivariate analyses. The increased mortality rate among patients with trigger events was particularly striking for nonmetastatic prostate cancer (1.3% vs 7.5%, adjusted odds ratio 1.96 [95% CI 1.49‐2.57]) and nonmetastatic colorectal cancer (4.1% vs 11.7%, 1.44 [1.19‐1.75]).

**Conclusions:**

The association between adverse event triggers and poor survival among a cohort of cancer patients supports the validity of a cancer‐specific, administrative claims‐based trigger tool.

## Graphical abstract

In a retrospective longitudinal cohort of 322 887 newly diagnosed patients with breast, colorectal, lung, or prostate cancer, there was increased one‐year mortality among patients who experienced oncology‐specific claims‐based triggers compared to patients without triggers. Increased mortality was observed across all cancer types, stratified by metastatic status, in unadjusted and multivariate analyses.

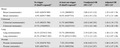

## 1 INTRODUCTION

Despite the complexity and potential toxicity of cancer‐specific therapy, there are few high‐quality studies characterizing the nature and extent of treatment‐related errors and injuries in clinical oncology.^1^, ^2^, ^3^ While toxicity assessment is deeply engrained in the cancer clinical trials tradition, much of this work seeks to identify the type and severity of adverse drug reactions inherent in novel therapies rather than injuries due to medical care that may follow a medical error or occur in a vulnerable host. A recent literature review concluded that no consistent methodology or large‐scale study allowed for an epidemiologically robust understanding of the extent of oncology treatment‐related errors and injuries by facility, location, or across the continuum of cancer care.^4^

A well‐established approach to identify treatment‐related complications uses “trigger” events (such as abnormal laboratory results, unplanned transfer to an intensive care unit, return to the operating room, or administration of antidote medications) to flag candidate adverse events for further review.^5^, ^6^, ^7^, ^8^, ^9^, ^10^ Unfortunately, due to the high expected symptom burden among cancer patients undergoing multi‐modal therapy, trigger tools evinced poor interrater reliability and low positive predictive values (PPVs) in multiple European studies.^11^, ^12^, ^13^, ^14^, ^15^, ^16^, ^17^, ^18^ However, researchers recently reported the successful development of 49 oncology‐specific triggers using clinical data from patients at Memorial Sloan Kettering Cancer Center (MSK) undergoing an initial course of cancer‐directed therapy. The PPV of the MSK triggers, using physician chart review as the gold standard, was satisfactory at 0.48 for adverse events and 0.18 for preventable events.^19^, ^20^

To extend this approach, we developed a set of oncology‐specific triggers that used International Classification of Diseases (ICD) and Current Procedural Terminology (CPT) codes to flag eligible cases in a large administrative database of commercial claims. We found high rates of oncology‐specific trigger events among a cohort of over 300,000 patients undergoing an initial course of cancer‐directed therapy for breast, colorectal, lung, or prostate cancer.^21^ The triggers affected 12% of patients with nonmetastatic disease and 39% of those with metastatic cancer. The burden was highest among patients with lung and colorectal cancer, and triggers were overrepresented among non‐whites, those with lower family incomes, and patients with lower educational attainment.

The goal of this study was to assess the construct validity of oncology‐specific triggers as a quality of care measure by examining the association between oncology triggers and mortality using administrative claims data. We hypothesized that patients who experienced trigger events would have higher mortality rates (controlling for relevant covariates) during an initial year of cancer‐directed therapy, and that the trigger‐mortality association would vary by trigger, cancer type, and metastatic status.

## 2 METHODS

### 2.1 Data

We accessed the OptumLabs^®^ Data Warehouse (OLDW), which includes deidentified administrative claims and electronic health record (EHR) data on over 200 million patients, including billing data for inpatient and ambulatory care for commercial and Medicare Advantage enrollees.^22^ It includes limited patient demographic information drawn from enrollment records. Socioeconomic status information in OLDW, including race/ethnicity, household income, and educational attainment, are imputed variables sourced from a national supplier of consumer marketing data. Mortality status is ascertained in OLDW through multiple sources including the Social Security Death Index, inpatient discharge status, and electronic medical records.

### 2.2 Subjects

We selected a cohort of patients undergoing an initial course of cancer‐directed therapy for breast, lung, colorectal, and prostate cancer, using ICD and CPT codes to identify patients who received surgery, radiation therapy, or chemotherapy (including oncolytic, hormonal, or targeted therapies) for a new diagnosis of breast, lung, colorectal, or prostate cancer from 1 January 2008 through 31 December 2014. Patients with cancer diagnoses or treatments in 2005‐2007 and those with cancer recurrence codes were excluded.^23^, ^24^, ^25^

We abstracted sociodemographic variables included in the OLDW including age, gender, race/ethnicity, insurance (commercial, Medicare Advantage), household income, and educational attainment, excluding subjects with male breast cancer and those under age 18. We also abstracted cancer diagnosis, cancer‐specific therapies, and associated diagnosis and treatment dates. We calculated a modified Charlson comorbidity index using an algorithm that excluded cancer as a comorbidity,^26^ and an algorithm developed and tested in the OLDW to identify metastatic cancer cases.^27^

### 2.3 Measurements

We defined a set of oncology‐specific triggers using ICD and CPT codes corresponding to 16 of the 23 highest (≥50%) PPV triggers from the MSK study. We constrained triggers to events that occurred within specified intervals following relevant exposures, and further assumed that triggered events had a discrete, limited duration. For example, blood transfusion could result from chemotherapy‐associated myelosuppression or intraoperative blood loss, but is an unlikely direct result of radiation therapy. Chemotherapy‐associated anemia requiring transfusion would be expected to develop over and persist for several weeks, while peri‐operative anemia would present within hours or days and be addressed within a similar interval. Treatment‐trigger causal and temporal relationships and trigger event durations were developed in collaboration with clinical oncologists.

### 2.4 Analyses

We characterized the cohort by sociodemographic and clinical attributes and cancer‐specific treatments, stratifying analyses by cancer type (breast, colorectal, lung, prostate) and metastatic status. We tabulated the number of patients who experienced at least one of each trigger type during the first year of cancer therapy by cancer type and metastatic status.

To assess the association of event triggers with mortality, we examined the number and percent of patients with each adverse event trigger who were alive or dead at the end of a one‐year period beginning with the date of the initial cancer‐directed therapy, performing separate analyses by cancer type and metastatic status.

We estimated adjusted odds ratios and 95% confidence intervals with logistic regression models stratified by cancer type and metastatic status to examine the relationship between the mortality outcome and types of triggers. Independent variables in the regression models controlled for sociodemographic and clinical attributes and the type of cancer‐directed therapy—factors that could affect the risk of death. Sociodemographic characteristics included age, gender, race/ethnicity, household income, and educational attainment. Clinical attributes included the Charlson index and treatment type (chemotherapy only, surgery only, radiation only, or multi‐modality therapy). Analyses also adjusted for the presence of other triggers.

In preliminary analyses, we noted slightly different trigger prevalence rates among patients with and without missing race/ethnicity, income, or educational attainment data for selected triggers. There was no consistent pattern, however. Concerned that our results could potentially be biased if trigger events were associated with incomplete collection or reporting of sociodemographic information, we used multiple imputation by chained equations, with predictive mean matching for numeric variables and logistic regression for binary variables, to impute multivariate missing data elements for patients with incomplete information. The model to impute missing information included the outcome variable, patient age, gender, race/ethnicity, household income, educational attainment, Charlson index, treatment type, and trigger indicators.^28^, ^29^ Average estimates and total variance from regression models fit to each of the imputed data sets were pooled following Rubin's rules.^30^ Multiple imputation imputes values for missing observations of a variable using other available information in the dataset. We imputed missing values under the assumption that data are missing at random (MAR), in other words, independent of the value of the variable and conditional on observed covariates. Ignoring the mechanism of "missing‐ness" by excluding records with any missing values could lead to biased estimates. While the MAR assumption is not fully testable, advantages of reduced risk in bias compared to a complete case analysis make MAR imputation the most reasonable approach. We understand that missing race/ethnicity, income, and education data reflects administrative practices at insurers that do not incorporate or prioritize collection of this information for enrollment. We are aware of no systematic biases introduced in this process.

In sensitivity analyses designed to assess the reliability of the model under different assumptions about patient enrollment, we examined the impact of excluding patients without a full year of continuous enrollment (and alive at last encounter) on the results. We knew from preliminary analyses that "no trigger" patients were more often lost to follow‐up than patients with triggers across all disease types. For example, 12.0% of "no trigger" patients with nonmetastatic breast cancer were lost to follow‐up compared to 10.4% among those with at least one trigger. Since there were more patients with incomplete follow‐up in the “no trigger” group, excluding these patients could increase the odds of finding higher mortality among “trigger” patients. In an alternate scenario, we assumed that lost‐to‐follow‐up patients had all died. This worst‐case scenario would bias the analysis toward the null hypothesis. The “exclusion” and “worst‐case” scenarios were compared to the primary analysis that included all lost‐to‐follow‐up patients and imputed missing one‐year mortality status.

Analyses were performed using SAS 9.4 for Windows (SAS Institute) and R 3.4.3 (The R Foundation). The Tufts Health Sciences Institutional Review Board (IRB) determined the project to be exempt from human subjects review.

## 3 RESULTS

### 3.1 Cohort characteristics

The final cohort included 322 887 patients (Table 1). The average age was 64. There was a higher percentage of men than women among patients with colorectal and lung cancer. Non‐whites accounted for at least 13% of the cohort, but race/ethnicity, household income, and education data were missing for about one‐third of the cohort. Twenty‐seven percent of patients had metastatic disease, with variation by cancer type.

**Table 1:** Cohort characteristics^a^

Characteristic	Breast	Colorectal	Lung	Prostate	Overall
N	124 253	52 383	51 311	94 940	322 887
Age [mean (SD)]	59.5 (12.1)	63.2 (12.5)	67.1 (10.5)	66.9 (9.1)	63.5 (11.6)
Sex [n (%)]					
Male	—	27 616 (52.7)	27 170 (53.0)	94 940 (100.0)	149 726 (46.4)
Female	124 253 (100.0)	24 767 (47.3)	24 141 (47.0)	—	173 161 (53.6)
Race/Ethnicity [n (%)]					
Missing/Unknown	37 198 (29.9)	18 961 (36.2)	19 432 (37.9)	32 741 (34.5)	108 332 (33.6)
Asian	2398 (1.9)	862 (1.6)	634 (1.2)	1019 (1.1)	4913 (1.5)
Black	9543 (7.7)	3847 (7.3)	3538 (6.9)	7920 (8.3)	24 848 (7.7)
Hispanic	5491 (4.4)	2360(4.5)	1271 (2.5)	3463 (3.6)	12 585 (3.9)
White	69 623 (56.0)	26 353 (50.3)	26 436 (51.5)	49 797 (52.5)	172 209 (53.3)
Annual household income [n (%)]					
Unknown	44 997 (36.2)	22 584 (43.1)	23 569 (45.9)	37 349 (39.3)	128 499 (39.8)
<$25K	15 627 (12.6)	6713 (12.8)	8845 (17.2)	10 733 (11.3)	41 918 (13.0)
$24K ‐ $149K	19 793 (15.9)	8511 (16.2)	8488 (16.5)	15 694 (16.5)	52 416 (16.2)
$150K ‐ 249K	23 057 (18.6)	8367 (16.0)	6551 (12.8)	17 314 (18.2)	55 289 (17.1)
$250K ‐ $499K	12 336 (9.9)	3964 (7.6)	2553 (5.0)	8514 (9.0)	27 367 (8.5)
$500K+	8513 (6.9)	2244 (4.3)	1305 (2.5)	5336 (5.6)	17 398 (5.4)
Education [n (%)]					
Missing/Unknown	34 322 (27.6)	17 925 (34.2)	18 449 (36.0)	30 698 (32.3)	101 394 (31.4)
Less than 12th grade	217 (0.2)	141 (0.3)	89 (0.2)	149 (0.2)	596 (0.2)
High school diploma	20 749 (16.7)	9891(18.9)	10 987 (21.4)	16 468 (17.3)	58 095 (18.0)
Less than bachelor degree	48 901 (39.4)	18 423 (35.2)	17 365 (33.8)	35 020 (36.9)	119 709 (37.1)
Bachelor degree plus	20 064 (16.1)	6003 (11.5)	4421 (8.6)	12 605 (13.3)	43 093 (13.3)
Insurance type [n (%)]					
Private insurance	99 932 (80.4)	42 456 (81.0)	37 196 (72.5)	69 486 (73.2)	249 070 (77.1)
Medicare Advantage	24 321 (19.6)	9927 (19.0)	14 115 (27.5)	25 454 (26.8)	73 817 (22.9)
Clinical characteristics					
Metastatic disease [n (%)]	26 791 (21.6)	18 671 (35.6)	30 169 (58.8)	10 800 (11.4)	86 431 (26.8)
Charlson index^b^ [mean (SD)]	2.0 (1.6)	2.8 (1.9)	3.6 (1.8)	2.8 (1.4)	2.6 (1.7)
Rehospitalized w/in 1 year [n (%)]	36 780 (29.6)	33 047 (63.1)	32 092 (62.5)	37 798 (39.8)	139 717 (43.3)
Hospital days [mean (SD)]	5.1 (8.8)	10.4 (13.9)	10.4 (12.9)	4.1 (7.9)	7.3 (11.4)
Treatment type [n (%)]					
Chemotherapy only	18 357 (14.8)	8724 (16.7)	11 501 (22.4)	18 830 (19.8)	57 412 (17.8)
Radiation only	11 407 (9.2)	1273 (2.4)	7260 (14.1)	21 253 (22.4)	41 193 (12.8)
Surgery only	22 952 (18.5)	22 770 (43.5)	6818 (13.3)	31 598 (33.3)	84 138 (26.1)
Multi‐modality	71 537 (57.6)	19 616 (37.4)	25 732 (50.1)	23 259 (24.5)	140 144 (43.4)

Note

Percentages may not add to 100% due to rounding.

^a^ Table 1 is reproduced from Weingart SN, Nelson J, Koethe B, et al Developing a cancer‐specific trigger tool to identify treatment‐related adverse events using administrative data. Cancer Med. 2020 Jan 3. https://doi.org/10.1002/cam4.2812.

^b^ NIH measure for cancer patients modification. https://healthcaredelivery.cancer.gov/seermedicare/considerations/calculation.html.

### 3.2 Trigger prevalence

As we have shown previously, trigger events were common among cohort members, affecting 19% of patients (Appendix A). Patients with colorectal and lung cancers were most likely to experience at least one trigger event. Patients with metastatic disease were particularly vulnerable to cancer‐specific triggers, affecting 41.4% of those with metastatic colorectal and 50.2% of those with metastatic lung cancers. The most commonly flagged triggers were for abnormal serum bicarbonate, blood transfusion, noncontrast chest CT following radiation therapy, hypoxemia, contact precautions, neutropenic fever, and abnormal serum potassium.

### 3.3 Overall mortality

Compared to patients with no oncology trigger, mortality among cancer patients with a treatment‐related trigger was substantial. As shown in Table 2, the overall one‐year mortality rate among patients with nonmetastatic cancer and at least one trigger event was higher than patients without a trigger across all cancers. For example, among patients with nonmetastatic breast cancer, 0.8% of no‐trigger patients died within one year, while 3.1% of those with at least one trigger died. The absolute increase in mortality was particularly striking for nonmetastatic colorectal cancer (4.1% vs 11.7%) and nonmetastatic lung cancer (18.1% vs 33.7%).

**Table 2:** Observed mortality rate and relative risk of death in the first year of cancer‐directed therapy among patients with and without at least one trigger event, bivariate and multivariate analyses by cancer type and metastatic status

	No trigger (N died/N exposed)^a^	At least one trigger (N died/N exposed)^a^	Unadjusted OR (95% CI)^b^	Adjusted OR (95% CI)^c^
Breast				
Breast (nonmetastatic)	0.8% (620/76 999)	3.1% (280/8923)	2.80 (2.43 3.22)	1.35 (1.03 1.76)
Breast (metastatic)	4.9% (829/17 080)	10.5% (810/7 733)	2.10 (1.91 2.32)	1.57 (1.31 1.88)
Colorectal				
Colorectal (nonmetastatic)	4.1% (983/23 788)	11.7% (683/5860)	2.65 (2.40 2.93)	1.44 (1.19 1.75)
Colorectal (metastatic)	15.7% (1556/9923)	22.7% (1623/7151)	1.53 (1.42 1.66)	1.38 (1.20 1.58)
Lung				
Lung (nonmetastatic)	18.1% (2274/12 544)	33.7% (2093/6202)	2.19 (2.05 2.34)	1.38 (1.21 1.58)
Lung (metastatic)	43.3% (5746/13 283)	47.7% (6531/13 693)	1.17 (1.12 1.23)	1.16 (1.07 1.26)
Prostate				
Prostate (nonmetastatic)	1.3% (916/70 762)	7.5% (351/4708)	4.56 (4.02 5.17)	1.96 (1.49 2.57)
Prostate (metastatic)	8.8% (685/7815)	20.1% (509/2536)	2.53 (2.24 2.87)	1.87 (1.47 2.39)

Abbreviations: CI, confidence interval; OR, odds ratio.

^a^ Denominator includes patients with a full year of continuous enrollment or death.

^b^ Unadjusted pooled odds ratio and 95% confidence interval using imputed outcome.

^c^ Pooled OR (95% CI) from multivariate imputed logistic regression controlling for treatment type, age, gender, race, education, income, Charlson index, and the presence of other triggers.

Oncology‐specific triggers were associated with increased one‐year mortality among patients with metastatic disease, albeit on a higher baseline mortality risk. As expected, the one‐year mortality rate for patients with metastases exceeded the rate of those with nonmetastatic disease for each cancer type. However, patients with metastatic disease and at least one trigger had poorer survival than those with metastatic disease of the same cancer type with no trigger. For example, among patients with metastatic prostate cancer, 8.8% of no‐trigger patients died within one year while 20.1% of those with at least one trigger died. In multivariate analyses involving the full cohort and controlling for potential confounders, experiencing at least one cancer‐specific trigger conferred increased odds of death within one year across all cancer types and for those with metastatic and nonmetastatic disease.

### 3.4 Trigger‐specific mortality

We replicated the “any trigger” analysis for each individual trigger, examining the relationship between specific triggers and one‐year mortality. The one‐year mortality rate varied by trigger and cancer type. Among patients with nonmetastatic cancer (Table 3), the mortality rate was higher among patients with 13 of the 14 cancer‐specific triggers where there were sufficient cases to calculate a death rate. (Cell frequencies <11 cases are not reported to avoid identifying unique patients.) Unexpectedly, lower one‐year mortality was associated with contact precautions among patients with nonmetastatic breast, colorectal, and lung cancers, suggesting that trigger events may be protective under certain circumstances. Institution of contact precautions may, for example, signal an appropriate response to early detection of an infectious complication.

**Table 3:** Observed mortality rate in the first year of cancer‐directed therapy among patients with and without at least one triggered event, by *NON*metastatic cancer type^a^

	Breast (n = 85 922)		Colorectal (n = 29 648)		Lung (n = 18 746)		Prostate (n = 75 470)	
	No trigger	Trigger	No trigger	Trigger	No trigger	Trigger	No trigger	Trigger
At least 1 trigger	0.8% (620/76 999)	3.1% (280/8923)	4.1% (983/23 788)	11.7% (683/5860)	18.1% (2274/12 544)	33.7% (2093/6202)	1.3% (916/70 762)	7.5% (351/4708)
Anticoagulation	1.0% (799/76 852)	< 11 deaths	5.3% (1541/28 886)	< 11 deaths	21.6% (3395/15 732)	23.9% (11/46)	1.9% (1074/57 440)	< 11 deaths
Bacteremia/positive blood culture	1.0% (771/76 665)	11.6% (29/250)	5.2% (1480/28 623)	20.0% (63/315)	21.3% (3305/15 528)	40.4% (101/250)	1.8% (1047/57 307)	15.3% (27/177)
Abnormal serum bicarbonate	1.0% (746/75 463)	3.7% (54/1452)	5.0% (1382/27 763)	13.7% (161/1175)	21.0% (3150/15 024)	34% (256/754)	1.8% (1024/56 289)	4.2% (50/1195)
Blood transfusion	0.9% (711/75 415)	5.9% (89/1500)	5.0% (1401/28 157)	18.2% (142/781)	19.7% (2720/13 829)	35.2% (686/1949)	1.8% (1005/57 105)	18.2% (69/379)
C. difficile positive	1.0% (780/76 645)	7.4% (20/270)	5.2% (1499/28 617)	13.7% (44/321)	21.3% (3322/15 574)	41.2% (84/204)	1.8% (1060/57 367)	12.0% (14/117)
Noncontrast chest CT following XRT	0.6% (301/47 026)	2.1% (16/768)	8.2% (423/5180)	22.7% (27/119)	30.7% (2332/7599)	31.7% (277/874)	1.2% (392/33 276)	5.0% (20/400)
Elevated creatinine	1.0% (895/85 806)	< 11 deaths	5.6% (1656/29 569)	< 11 deaths	23.3% (4339/18 637)	25.7% (28/109)	1.7% (1260/75 412)	< 11 deaths
Low oximetry	1.0% (809/85 009)	10.0% (91/913)	5.2% (1496/28 789)	19.8% (170/859)	20.8% (3407/16 415)	41.2% (960/2331)	1.5% (1142/74 490)	12.8% (125/980)
Contact precautions/ isolation	1.3% (484/36 661)	0.9% (22/2335)	7.1% (719/10 148)	5.0% (22/436)	29.2% (2855/9783)	20.8% (133/640)	3.4% (912/26 963)	5.3% (18/340)
Nasogastric tube	0.7% (417/59 977)	< 11 deaths	4.2% (985/23 649)	< 11 deaths	8.3% (596/7198)	< 11 deaths	0.6% (192/33 821)	< 11 deaths
Nephrology consult	1.0% (877/85 686)	9.7% (23/236)	5.3% (1557/29 208)	24.8% (109/440)	23.0% (4258/18 489)	42.4% (109/257)	1.6% (1222/74 948)	8.6% (45/522)
Neutropenic fever	1.2% (448/37 448)	3.7% (58/1548)	6.7% (678/10 109)	13.3% (63/475)	28.1% (2730/9713)	36.3% (258/710)	3.3% (906/27 137)	14.5% (24/166)
Percutaneous drain	0.7% (416/59 962)	< 11 deaths	4.2% (980/23 570)	< 11 deaths	8.3% (591/7159)	< 11 deaths	0.6 % (191/33 759)	< 11 deaths
Abnormal serum potassium	1.0% (728/74 908)	3.6% (72/2007)	5.0% (1332/26 750)	9.6% (211/2188)	20.6% (2984/14 484)	32.6% (422/1294)	1.8% (991/56 406)	7.7% (83/1078)
Pressure ulcer	0.7% (408/59 770)	< 11 deaths	4.0% (937/23 477)	18.8% (52/276)	8.1% (578/7128)	26% (19/73)	0.5% (184/33 759)	< 11 deaths
Return to OR/IR	0.7% (415/59 925)	< 11 deaths	4.1% (955/23 167)	5.8% (34/586)	8.2% (590/7171)	< 11 deaths	0.6% (190/33 638)	< 11 deaths

Abbreviations: CT, computed tomography; IR interventional radiology; OR, operating room; XRT, radiation therapy.

^a^ Observed mortality rate (n died/n exposed); excludes patients lost to follow‐up.

Table 4 displays mortality rates, by trigger, among patients with metastatic disease. Mortality rates were higher among patients with a cancer‐specific trigger than among patients with no trigger with several exceptions (eg, anticoagulation and return to the operating room triggers among colorectal cancer patients, and elevated creatinine among patients with lung cancer). Like the nonmetastatic cancer cases, contact precautions among patients with metastatic disease were associated with lower mortality rates across all cancer types.

**Table 4:** Observed mortality rate in the first year of cancer‐directed therapy among patients with and without at least one triggered event, by *METASTATIC* cancer type^a^

	Breast (n = 24 813)		Colorectal (n = 17 074)		Lung (n = 26 976)		Prostate (n = 10 351)	
	No trigger	Trigger	No trigger	Trigger	No trigger	Trigger	No trigger	Trigger
At least 1 trigger	4.9% (829/17 080)	10.5% (810/7 733)	15.7% (1 556/9 923)	22.7% (1 623/7 151)	43.3% (5 746/13 283)	47.7% (6 531/13 693)	8.8% (685/7 815)	20.1% (509/2 536)
Anticoagulation	6% (1412/23 576)	< 11 deaths	18.1% (2993/16 581)	17.4% (15/86)	41.8% (9838/23 523)	43.3% (39/90)	11.2% (1056/9395)	< 11 deaths
Bacteremia/positive blood culture	5.8% (1361/23 281)	15% (52/346)	17.7% (2860/16 141)	28.1% (148/526)	41.6% (9585/23 052)	52% (292/561)	11.1% (1030/9264)	18.5% (27/146)
Abnormal serum bicarbonate	5.6% (1257/22 403)	12.7% (156/1224)	17.6% (2657/15 104)	22.5% (351/1563)	41.6% (9101/21 888)	45.0% (776/1725)	10.9% (959/8809)	16.3% (98/601)
Blood transfusion	5.2% (1119/21 529)	14.0% (294/2098)	16.8% (2528/15 039)	29.5% (480/1628)	39.9% (7236/18 115)	48.0% (2641/5498)	9.8% (849/8692)	29.0% (208/718)
C. difficile positive	5.9% (1380/23 381)	13.4% (33/246)	17.9% (2905/16 257)	25.1% (103/410)	41.6% (9645/23 182)	53.8% (232/431)	11.1% (1040/9346)	26.6% (17/64)
Noncontrast chest CT following XRT	5.2% (792/15 211)	14.1% (67/475)	18.1% (841/4657)	29.8% (61/205)	49.1% (8038/16 367)	46.6% (1000/2147)	12.8% (519/4039)	20.4% (43/211)
Elevated creatinine	6.6% (1630/24 681)	< 11 deaths	18.5% (3139/16 930)	27.8% (40/144)	45.6% (12171/26 697)	38.0% (106/279)	11.5% (1181/10 312)	33.3% (13/39)
Low oximetry	5.9% (1406/23 802)	23.0% (233/1011)	17.7% (2842/16 038)	32.5% (337/1036)	43.2% (9734/22 536)	57.3% (2543/4440)	10.8% (1066/9889)	27.7% (128/462)
Contact precautions/ isolation	6.4% (1213/18 977)	4.3% (85/1993)	17.1% (2306/13 453)	12.8% (110/860)	43.6% (8890/20 407)	35.9% (639/1781)	12.0% (992/8270)	8.4% (25/299)
Nasogastric tube	2.7% (382/14 223)	< 11 deaths	13.9% (1340/9612)	< 11 deaths	22.1% (1042/4715)	< 11 deaths	6.2% (121/1954)	< 11 deaths
Nephrology consult	6.4% (1558/24 476)	24.0% (81/337)	18.3% (3018/16 523)	29.2% (161/551)	45.4% (11967/26 381)	52.1% (310/595)	11.4% (1148/10 073)	16.5% (46/278)
Neutropenic fever	6.0% (1174/19 553)	8.8% (124/1417)	16.5% (2231/13 484)	22.3% (185/829)	42.9% (8817/20 543)	43.3% (712/1645)	11.6% (970/8349)	21.4% (47/220)
Percutaneous drain	2.6% (375/14 208)	< 11 deaths	13.8% (1299/9445)	20.9% (48/230)	22.0% (1033/4695)	< 11 deaths	6.2% (120/1949)	< 11 deaths
Abnormal serum potassium	5.4% (1158/21 560)	12.3% (255/2067)	17.2% (2362/13 710)	21.8% (646/2957)	41.3% (8385/20 317)	45.3% (1492/3296)	10.7% (942/8771)	18.0% (115/639)
Pressure ulcer	2.7% (375/14 145)	< 11 deaths	13.8% (1315/9536)	23.0% (32/139)	21.9% (1024/4677)	46.2% (18/39)	6.2% (121/1943)	< 11 deaths
Return to OR/IR	2.7% (382/14 193)	< 11 deaths	14.0% (1304/9324)	12.3% (43/351)	22.0% (1031/4695)	100% (11/11)	6.2% (121/1946)	< 11 deaths

Abbreviations: CT, computed tomography; IR interventional radiology; OR, operating room; XRT, radiation therapy.

^a^ Observed mortality rate (n died/n exposed); excludes patients lost to follow‐up.

We found a similar pattern in multivariate analyses, modeling the independent relationship of each trigger to mortality by cancer type and metastatic status. As shown in Table 5, bacteremia, blood transfusion, hypoxemia, and nephrology consultation were triggers most commonly associated with increased odds of death. In contrast, contact precautions were associated with reduced odds of death across most cancer types.

**Table 5:** Adjusted odds ratios and 95% confidence intervals of death in the first year of cancer‐directed therapy among patients with and without a specific triggered event, by cancer type and metastatic status, multivariate analysis^a^

	Breast		Colorectal		Lung		Prostate	
	Nonmetastatic (n = 97 462)	Metastatic (n = 26 791)	Nonmetastatic (n = 33 712)	Metastatic (n = 18 671)	Nonmetastatic (n = 21 142)	Metastatic (n = 30 169)	Nonmetastatic (n = 84 140)	Metastatic (n = 10 800)
At least one trigger	1.35 (1.03, 1.76)*	1.57 (1.31, 1.88)***, a	1.44 (1.19, 1.75)***, a	1.38 (1.20, 1.58)***, a	1.38 (1.21, 1.58)***, a	1.16 (1.07, 1.26)***, a	1.96 (1.49, 2.57)***, a	1.87 (1.47, 2.39)***, a
Anticoagulation	0.62 (0.09, 4.52)	0.19 (0.02, 1.51)	0.33 (0.08, 1.41)	0.79 (0.45, 1.40)	0.66 (0.32, 1.38)	0.96 (0.62, 1.48)	0.00 (0.00, >50)	0.25 (0.03, 1.88)
Bacteremia/ positive blood culture	2.55 (1.62, 4.00)***, a	1.16 (0.84, 1.61)	1.80 (1.33, 2.44)***, a	1.27 (1.03, 1.56)*	1.53 (1.14, 2.04)**	1.27 (1.06, 1.52)**	2.19 (1.36, 3.54)**	0.87 (0.55, 1.39)
Abnormal serum bicarbonate	1.22 (0.89, 1.69)	1.15 (0.94, 1.42)	1.38 (1.12, 1.70)**	1.02 (0.89, 1.18)	1.09 (0.91, 1.29)	0.97 (0.87, 1.07)	0.55 (0.40, 0.77)***, a	0.85 (0.65, 1.10)
Blood transfusion	2.20 (1.68, 2.88)***, a	1.48 (1.25, 1.75)***, a	1.68 (1.35, 2.10)***, a	1.57 (1.37, 1.81)***, a	1.04 (0.91, 1.20)	1.14 (1.05, 1.22)***, a	2.59 (1.87, 3.60)***, a	2.12 (1.69, 2.67)***, a
C. difficile positive	1.70 (1.02, 2.84)*	1.19 (0.80, 1.77)	1.13 (0.80, 1.60)	1.10 (0.87, 1.40)	1.30 (0.96, 1.77)	1.46 (1.20, 1.79)***, a	1.27 (0.69, 2.36)	1.70 (0.91, 3.18)
Noncontrast chest CT following XRT	1.34 (0.74, 2.45)	1.51 (1.10, 2.06)*	1.48 (0.89, 2.46)	1.27 (0.91, 1.78)	0.75 (0.63, 0.89)***, a	0.80 (0.72, 0.90)***, a	1.22 (0.71, 2.09)	0.92 (0.62, 1.36)
Elevated creatinine	1.68 (0.65, 4.35)	0.50 (0.24, 1.01)	1.12 (0.55, 2.31)	1.23 (0.83, 1.80)	0.78 (0.50, 1.24)	0.70 (0.54, 0.89)**	2.07 (0.85, 5.07)	2.46 (1.14, 5.32)*
Hypoxemia/low oximetry	2.50 (1.86, 3.35)***, a	2.15 (1.78, 2.60)***, a	1.86 (1.49, 2.32)***, a	1.64 (1.41, 1.91)***, a	1.56 (1.37, 1.79)***, a	1.57 (1.45, 1.70)	1.64 (1.23, 2.18)***, a	1.55 (1.18, 2.02)**
Contact precautions/ isolation	0.59 (0.38, 0.91)*	0.63 (0.49, 0.81)***, a	0.67 (0.42, 1.05)	0.61 (0.49, 0.75)***, a	0.52 (0.42, 0.64)***, a	0.66 (0.59, 0.73)***, a	0.87 (0.51, 1.49)	0.41 (0.26, 0.66)***, a
Nephrology consult	2.48 (1.51, 4.09)***, a	2.05 (1.54, 2.74)***, a	2.81 (2.14, 3.68)***, a	1.32 (1.07, 1.63)**	2.01 (1.52, 2.67)***, a	1.35 (1.13, 1.60)***, a	1.37 (0.96, 1.96)	0.87 (0.61, 1.25)
Neutropenic fever	1.58 (1.14, 2.18)**	1.09 (0.88, 1.35)	1.12 (0.83, 1.51)	1.02 (0.84, 1.22)	1.14 (0.95, 1.37)	0.88 (0.79, 0.98)*	1.48 (0.90, 2.44)	1.29 (0.90, 1.86)
Percutaneous drain	1.28 (0.12, 13.23)	14.62 (4.85, 44.01)***, a	0.80 (0.41, 1.56)	1.54 (1.08, 2.19)*	1.07 (0.43, 2.68)	2.32 (0.90, 5.98)	0.60 (0.09, 4.11)	2.54 (0.52, 12.40)
Abnormal serum potassium	1.05 (0.78, 1.41)	1.07 (0.89, 1.28)	0.87 (0.72, 1.04)	1.05 (0.93, 1.19)	1.02 (0.88, 1.18)	1.00 (0.92, 1.09)	1.02 (0.77, 1.35)	0.89 (0.68, 1.15)
Pressure ulcer	1.48 (0.74, 2.93)	0.96 (0.40, 2.29)	1.58 (1.10, 2.26)*	0.96 (0.62, 1.47)	1.88 (1.05, 3.37)*	2.10 (1.08, 4.09)*	2.05 (0.90, 4.65)	0.29 (0.04, 2.27)
Return to OR/IR	1.64 (0.38, 7.03)	0.01 (0.00, >50)	0.99 (0.67, 1.44)	0.77 (0.56, 1.08)	1.94 (0.81, 4.61)	2.79 (1.13, 6.89)*	0.72 (0.16, 3.32)	0.68 (0.08, 5.48)

Abbreviations: CT, computed tomography; IR interventional radiology; OR operating room; XRT, radiation therapy.

^a^ Pooled odds ratios and 95% CI from multivariate imputed logistic regression controlling for treatment type, age, gender, race, education, income, Charlson index, and the presence of other triggers.

*** *P* < .001,

** *P* < .01,

* *P* < .05.

### 3.5 Sensitivity analyses

As there was a higher percent of patients lost to follow up among those with no trigger compared patients with at least one, it was possible that exclusion of lost‐to‐follow‐up patients might increase the apparent mortality risk in the cohort. We performed sensitivity testing to examine the durability of results based on the exclusion, inclusion, or imputation of mortality outcomes of patients lost to follow‐up during the initial year of cancer‐directed therapy. We found, for example, that the adjusted odds of death in the first treatment year for metastatic breast cancer patients with at least one trigger event was 1.57 using multiple imputation. This value was between 1.68 (lost cases excluded) and 1.21 (lost cases presumed dead), consistent with our hypothesis. This pattern was consistent in sensitivity analysis with each disease type and by metastatic status, as shown in Appendix B. Appendix B also shows that lost‐to‐follow‐up patients comprised 4%‐12% of the sample. Reassured about the consistency of the analyses, we presorted adjusted odds ratios and confidence intervals in Tables 2 and 5 using imputed mortality outcomes that include patients lost to follow‐up.

## 4 DISCUSSION

In this retrospective cohort study of 322 887 patients with breast, colorectal, lung and prostate cancer treated for an initial course of cancer‐directed therapy, patients with at least one cancer‐specific trigger had a substantially higher mortality risk than patients with no trigger. This relationship was robust across cancer types and metastatic status in logistic regression models controlling for multiple potential confounders. Patients who experienced treatment‐related triggers were at substantially higher risk than those with none. Individual triggers, including bacteremia, blood transfusion, hypoxemia, nephrology consultation, neutropenic fever, pressure ulcers, and return to the operating room or interventional radiology suite, were strongly associated with poor one‐year survival.

To assess the validity of cancer‐specific adverse event triggers, we hypothesized that patients with any adverse event trigger would be more likely to die than those with no trigger, other things equal. This effect should be evident among a cancer cohort as these are patients with serious illnesses undergoing toxic therapies and may have relatively little physiologic reserve to overcome an iatrogenic injury. The convergence of triggers, which denotes treatment‐related adverse events, and mortality would support the construct validity of event triggers as quality of care measures.

Previous validation studies of adverse event trigger tools relied on concurrent or retrospective medical record review.^7^, ^8^, ^9^, ^31^ Expert clinicians judged whether a trigger indicated that the patient suffered harm. Validation rates varied by trigger, with PPVs of 17%‐45% for groups of triggers comprising a trigger tool using physician chart review as the gold standard. Lipitz‐Snyderman and colleagues used this approach to derive the triggers employed in the current study, selecting from among 49 candidate triggers with an aggregate PPV of 44.8% those 16 items with individual PPVs exceeding 50%.^19^

We are aware of no previous research applying trigger tools to administrative data. However, Iezzoni, Romano, and others have used diagnosis and procedure codes to flag potential surgical and procedural complications in what have become the US Agency for Healthcare Research and Quality's Patient Safety Indicators (PSIs).^32^, ^33^, ^34^ Validation of the PSIs relied initially on demonstration of higher‐than‐expected mortality rates among patients with PSI flags compared to patients without, correlation with other quality metrics, and retrospective chart review of flagged cases.^35^, ^36^, ^37^, ^38^ While many PSI indicators have been criticized for lack of or poor validation based on medical record review, advocates argue that rate‐based metrics (such as PSI‐3 "failure to rescue") can be validated based on correlation with mortality and other outcome metrics.^39^

Medical record review is generally the gold standard for evaluating the validity of a proposed quality measure. Our adverse event triggers were derived from medical record review and then encoded using diagnosis and procedure codes for use with billing data. In future research, claims‐based triggers will need to be "reverse" validated to ensure triggers appropriately identify problematic cases. Several groups have described the use of triggers, embedded in electronic medical record systems, to flag cases with potential adverse events for investigation and mitigation.^40^, ^41^, ^42^, ^43^, ^44^ That said, there is significant potential benefit from trigger tools that use administrative data, as these instruments can be used to examine the performance within and across health care organizations, networks, and regions to characterize patient safety outcomes. This is especially important in cancer care, where institution and population‐level quality metrics are sparse.^45^ A measure set that draws from administrative data and spans the continuum of cancer care over an initial course of therapy may afford clinical leaders, researchers, and policy makers a useful tool for assessing disease‐ and treatment‐specific harm among extraordinarily vulnerable patients.

This research is subject to several limitations. The OLDW includes information about commercial and Medicare Advantage patients and our findings therefore may not be generalizable to a Medicare Fee for Service or Medicaid cohort. Claims‐based algorithms may fail to accurately distinguish patients with late recurrences and to characterize those with metastatic disease, although we sought to minimize this problem by drawing on well‐validated coding algorithms. Given the burden of disease‐related morbidity in cancer care, there is expected confounding of adverse events attributed to disease and to treatment. We attempted to address this inherent challenge by linking treatment exposure to triggered event by type of exposure, timing of event relative to exposure, and duration of event. While this approach improved the likelihood that a given trigger was caused by treatment, perfect attribution of trigger to treatment would require expert chart review. Certain important clinical variables, such as performance status (ie, the type of assistance required to perform normal activities), were unavailable to the research team and could affect mortality risk. Although we may have underestimated mortality rates given the well‐known limitations of the Social Security Death Master File, use of mortality status in OLDW mitigates some concern by drawing on multiple data sources.

In sum, this commercial claims‐based study found a consistent association between cancer‐specific adverse event triggers and poor survival among a large cohort of patients with breast, colorectal, lung, and prostate cancer. This finding supports the validity of a cancer‐specific trigger tool for measuring quality of care in oncology, although further research is needed to replicate these findings.

## CONFLICT OF INTEREST

The authors report no conflict of interest with this work.

## AUTHOR CONTRIBUTIONS

All of the authors attest that they have made substantial contributions to the conception and design, acquisition of data, and analysis and interpretation of data; have been involved in drafting the manuscript or revising it critically for important intellectual content; have given final approval of the version to be published. Each author participated sufficiently in the work to take public responsibility for appropriate portions of the content; and agreed to be accountable for all aspects of the work in ensuring that questions related to the accuracy or integrity of any part of the work are appropriately investigated and resolved.

## Data Availability

The data that support the findings of this study are available from OptumLabs^®^. Restrictions apply to the availability of these data, which were used under license for this study.

## Appendix A . Trigger prevalence within 1 year, by cancer type and metastatic status

**Table:**

Trigger	Nonmetastatic cancer n = 236 456				Metastatic cancer n = 86 431			
	Breast n = 97 462	Colorectal n = 33 712	Lung n = 21 142	Prostate n = 84 140	Breast n = 26 791	Colorectal n = 18 671	Lung n = 30 169	Prostate n = 10 800
Any trigger	10.2%	19.2%	32.8%	5.9%	30.9%	41.4%	50.2%	24.6%
Anticoagulation	0.1%	0.2%	0.3%	0.1%	0.2%	0.5%	0.4%	0.2%
Bacteremia/positive blood culture	0.3%	1.1%	1.6%	0.3%	1.4%	3.1%	2.4%	1.6%
Abnormal serum bicarbonate	1.8%	3.9%	4.7%	1.9%	5.0%	9.3%	7.3%	6.3%
Blood transfusion	1.9%	2.6%	12.2%	0.6%	8.8%	9.6%	23.2%	7.6%
C. difficile positive	0.3%	1.1%	1.3%	0.2%	1.1%	2.5%	1.8%	0.7%
Noncontrast chest CT following XRT	1.6%	2.1%	10.3%	1.1%	3.0%	4.1%	11.3%	4.9%
Elevated creatinine	0.1%	0.3%	0.6%	0.1%	0.5%	0.8%	1.0%	0.4%
Hypoxemia/ low oximetry	1.0%	2.8%	12.2%	1.2%	4.0%	5.9%	16.3%	4.6%
Contact precautions/isolation	6.0%	4.1%	6.2%	1.2%	9.5%	6.0%	8.0%	3.6%
Nasogastric tube	<11 events	0.5%	<11 events	0.1%	<11 events	0.6%	<11 events	<11 events
Nephrology consult	0.3%	1.4%	1.3%	0.6%	1.3%	3.1%	2.1%	2.6%
Neutropenic fever	4.0%	4.5%	6.7%	0.6%	6.8%	5.7%	7.4%	2.6%
Percutaneous drain	0%	0.8%	0.6%	0.3%	0.1%	2.4%	0.5%	0.6%
Abnormal serum potassium	2.5%	7.3%	8.1%	1.8%	8.7%	17.7%	13.9%	6.9%
Press ulcer	0.3%	1.2%	1.0%	0.2%	0.6%	1.5%	0.9%	0.8%
Return to OR or IR	0.1%	2.4%	0.4%	0.6%	0.2%	3.7%	0.4%	0.7%

*Note:* Values shown in the table are prevalence rates of patients with a trigger within an exposure window among those with the relevant treatment exposure.

Abbreviations: CT, computed tomography; IR interventional radiology**; **OR, operating room; XRT, radiation therapy.

## Appendix B . Observed relative risk of death in the first year of cancer‐directed therapy among patients with and without at least one trigger event, multivariate analyses by cancer type and metastatic status: sensitivity analysis using alternate assumptions about cases lost to follow‐up

**Table:**

	LTF excluded		LTF included (imputed or died)		
	N	LTF excluded OR (95% CI)^a^	N	LTF imputed OR (95% CI)^a^	LTF died (worst case) OR (95% CI)^a^
Breast					
Breast (nonmetastatic)	85 922	1.64 (1.22, 2.19)	97 462	1.35 (1.03, 1.76)	1.03 (0.90, 1.19)
Breast (metastatic)	24 813	1.68 (1.40, 2.02)	26 791	1.57 (1.31, 1.88)	1.21 (1.05, 1.38)
Colorectal					
Colorectal (nonmetastatic)	29 648	1.55 (1.28, 1.88)	33 712	1.44 (1.19, 1.75)	1.10 (0.96, 1.25)
Colorectal (metastatic)	17,074	1.43 (1.25, 1.63)	18 671	1.38 (1.20, 1.58)	1.16 (1.03, 1.30)
Lung					
Lung (nonmetastatic)	18 746	1.42 (1.24, 1.62)	21 142	1.38 (1.21, 1.58)	1.27 (1.13, 1.42)
Lung (metastatic)	26 976	1.18 (1.08, 1.28)	30 169	1.16 (1.07, 1.26)	1.13 (1.04, 1.22)
Prostate					
Prostate (nonmetastatic)	75 470	2.21 (1.68, 2.91)	84 140	1.96 (1.49, 2.57)	0.92 (0.75, 1.13)
Prostate (metastatic)	10 351	1.95 (1.53, 2.48)	10 800	1.87 (1.47, 2.39)	1.65 (1.33, 2.04)

Abbreviations: CI, confidence interval; LTF, lost to follow‐up; OR, odds ratio.

^a^ Pooled OR (95% CI) from multivariate logistic regression controlling for treatment type, age, gender, race, education, income, Charlson index, and the presence of other triggers.

## References

[1] Gandhi TK , Bartel SB , Shulman LN , et al. Medication safety in the ambulatory chemotherapy setting. Cancer. 2005;104:2477‐2483. 16245353 10.1002/cncr.21442

[2] Walsh KE , Dodd KS , Seetharaman K , et al. Medication errors among adults and children with cancer in the outpatient setting. J Clin Oncol. 2009;27:891‐896. 19114695 10.1200/JCO.2008.18.6072

[3] Lipitz‐Snyderman A , Pfister D , Classen D , et al. Preventable and mitigable adverse events in cancer care: measuring risk and harm across the continuum. Cancer. 2017;123:4728‐4736. 28817180 10.1002/cncr.30916 PMC7539686

[4] Weingart SN , Zhu L , Sweeney M , Hassett M . Chemotherapy medication errors. Lancet Oncol. 2018;19:e191‐e199. 29611527 10.1016/S1470-2045(18)30094-9

[5] IHI trigger tool for measuring adverse events. Available at: http://www.ihi.org/resources/Pages/Tools/IHIGlobalTriggerToolforMeasuringAEs.aspx. Accessed August 18, 2019.

[6] Classen DC , Jaser L , Budnitz DS . Adverse drug events among hospitalized Medicare pateints: epidemiology and national estimates from a new approach to surveillance. Jt Comm J Qual Safe. 2010;36:12‐21. 10.1016/s1553-7250(10)36003-x 20112660

[7] Sharek PJ , Horbar JD , Mason W , et al. Adverse events in the neonatal intensive care unit: development, testing, and findings of an NICU‐focused trigger tool to identify harm in North American NICUs. Pediatrics. 2006;116:1332‐1340. 10.1542/peds.2006-0565 17015521

[8] Unbeck M , Lindemalm S , Nydert P , et al. Validation of triggers and development of a pediatric trigger tool to identify adverse events. BMC Health Serv Res. 2014;14:655. 25527905 10.1186/s12913-014-0655-5 PMC4300839

[9] Stockwell DC , Bisarya H , Classen DC , et al. A trigger tool to detect harm in pediatric inpatient settings. Pediatrics. 2015;135:1036‐1042. 25986015 10.1542/peds.2014-2152

[10] Hibbert PD , Molloy CJ , Hooper TD , et al. The application of the Global Trigger Tool: a systematic review. Int J Qual Health Care. 2016;28:640‐649. 27664822 10.1093/intqhc/mzw115

[11] NHS Wales . How to use trigger tools (Appendix 3). Available at: http://www.1000livesplus.wales.nhs.uk/sitesplus/documents/1011/T4I%20%284%29%20How%20to%20use%20Trigger%20Tools%20%28Feb%202011%29%20Web.pdf. Accessed August 18, 2019.

[12] Mattsson TO , Knudsen JL , Brixen K , Herrstedt J . Does adding an appended oncology module to the Global Trigger Tool increase its value? Int J Qual Health Care. 2014;26:553‐560. 25080549 10.1093/intqhc/mzu072

[13] Bjørn B , Anhøj J , Østergaard M , Kodal AM , von Plessen C . Test‐retest reliability of an experienced global trigger tool review team. J Patient Saf, 2017; epub ahead of print. 10.1097/PTS.0000000000000433. 29023303

[14] Call RJ , Burlison JD , Robertson JJ , et al. Adverse drug detection in pediatric oncology and hematology patients: using medication triggers to identify patient harm in a specialized pediatric patient population. J Pediatr. 2014;165:447‐452.E4. 24768254 10.1016/j.jpeds.2014.03.033 PMC4145034

[15] Lipczak H , Neckelmann K , Steding‐Jessen M , Jakobsen E , Knudsen JL . Uncertain added value of Global Trigger Tool for monitoring of patient safety in cancer care. Dan Med Bul. 2011;58:1‐5. 22047933

[16] Mattsson TO , Knudsen JL , Lauritsen J , Broxen K , Herrstedt J . Assessment of the global trigger tool to measure, monitor and evaluate patient safety in cancer patients: reliability concerns are raised. BMJ Qual Saf. 2013;22:571‐579. 10.1136/bmjqs-2012-001219 23447657

[17] Hébert G , Netzer F , Kouakou SL , Lemare F , Minvielle E . Development of a 'ready‐to‐use' tool that includes preventability, for the assessment of adverse events in oncology. Int J Clin Pharm. 2018;40:376‐385. 29446003 10.1007/s11096-017-0542-3

[18] Hébert G , Netzer F , Ferrua M , Ducreux M , Lemare F , Minvielle E . Evaluating iatrogenic prescribing: development of an oncology‐focused trigger tool. Eur J Cancer. 2015;51:427‐435. 25549531 10.1016/j.ejca.2014.12.002

[19] Lipitz‐Snyderman A , Classen D , Pfister DG , et al. Performance of a trigger tool for identifying adverse events in oncology. J Oncol Pract. 2017;13:223‐230. 10.1200/JOP.2016.016634 PMC5482407 28095173

[20] Lipitz‐Snyderman A , Weingart SN , Anderson C , et al. ReCAP: Detection of potentially avoidable harm in oncology from patient medical records. J Oncol Pract. 2016;12(178–9):e224‐e230. 10.1200/JOP.2015.006874 PMC5486447 26869656

[21] Weingart SN , Nelson J , Koethe B , et al. Developing a cancer‐specific trigger tool to identify treatment‐related adverse events using administrative data. Cancer Med. 2020;9:1462‐1472. 31899856 10.1002/cam4.2812 PMC7013078

[22] OptumLabs . OptumLabs and OptumLabs Data Warehouse (OLDW) descriptions and citation. Cambridge, MA, May 2019.

[23] Hassett MJ , Uno H , Cronin AM , Carroll NM , Hornbrook MC , Ritzwoller D . Detecting lung and colorectal cancer recurrence using structured clinical/administrative data to enable outcomes research and population health management. Med Care. 2017;55:e88‐e98. 29135771 10.1097/MLR.0000000000000404 PMC4732933

[24] Ritzwoller DP , Hassett MJ , Uno H , et al. validation, and dissemination of a breast cancer recurrence detection and timing informatics algorithm. J Natl Cancer Inst. 2018;110(3):273–281. 29873757 10.1093/jnci/djx200 PMC5972574

[25] Hassett MJ , Ritzwoller DP , Taback N , et al. Validating billing/encounter codes as indicators of lung, colorectal, breast, and prostate cancer recurrence using two large contemporary cohorts. Med Care. 2014;52:e65‐e73. 23222531 10.1097/MLR.0b013e318277eb6f PMC3600389

[26] National Cancer Institute . Comorbidity SAS Macro (2014). Available at: https://healthcaredelivery.cancer.gov/seermedicare/considerations/macro-2014.html. Accessed August 18, 2019.

[27] Whyte JL , Engel‐Nitz NM , Teitelbaum A , Rey GG , Kallich JD . An evaluation of algorithms for identifying metastatic breast, lung, or colorectal cancer in administrative claims data. Med Care. 2015;53:e49‐e57. 23524464 10.1097/MLR.0b013e318289c3fb

[28] van Buuren S , Brand JPL , Groothuis‐Oudshoorn CGM , Rubin DB . Fully conditional specification in multivariate imputation. J Stat Comput Simul. 2006;76:1049‐1064.

[29] van Buuren S , Groothuis‐Oudshoorn K . MICE: multivariate imputation by chained equations in R. J Stat Softw. 2011;45:1‐67.

[30] Rubin DB . Multiple Imputation for Nonresponse in Surveys. New York, NY: John Wiley and Sons; 1987.

[31] Lim D , Melucci J , Rizer MK , Prier BE , Weber RJ . Detection of adverse drug events using an electronic trigger tool. Am J Health Syst Pharm. 2016;73:S112‐S120. 27543596 10.2146/ajhp150481

[32] McDonald KM , Romano PS , Geppert J , et al. eds. Measures of Patient Safety Based on Hospital Administrative Data: The Patient Safety Indicators. Rockville, MD: Agency for Healthcare Research and Quality (US); 2002. Report No.: 02–0038. 20734521

[33] Agency for Healthcare Research and Quality . Patient Safety Indicators Overview. Available at: https://www.qualityindicators.ahrq.gov/modules/psi_overview.aspx. Accessed August 18, 2019.

[34] Iezzoni LI , Daley J , Heeren T , et al. Identifying complications of care using administrative data. Med Care. 1994;32:700‐715. 8028405 10.1097/00005650-199407000-00004

[35] Ramanathan R , Leavell P , Stockslager G , Mays C , Harvey D , Duane TM . Validity of agency for healthcare research and quality patient safety indicators at an academic medical center. Am Surg. 2013;79:578‐582. 23711266

[36] Henderson KE , Aj R , Reichley RM , et al. Clinical validation of the AHRQ postoperative venous thromboembolism patient safety indicator. Jt Comm J Qual Patient Saf. 2009;35:370‐376. 19634805 10.1016/s1553-7250(09)35052-7

[37] Romano PS , Mull HJ , Rivard PE , et al. Validity of selected AHRQ patient safety indicators based on VA National Surgical Quality Improvement Program data. Health Serv Res. 2009;44:182‐204. 18823449 10.1111/j.1475-6773.2008.00905.x PMC2669628

[38] Rosen AK , Itani KM , Cevasco M , et al. Validating the patient safety indicators in the Veterans Health Administration: do they accurately identify true safety events? Med Care. 2012;50:74‐85. 21993057 10.1097/MLR.0b013e3182293edf

[39] Winters BD , Bharmal A , Wilson RF , et al. Validity of the Agency for Healthcare Research and Quality Patient Safety Indicators and the Centers for Medicare and Medicaid Hospital‐acquired Conditions: A systematic review and meta‐analysis. Med Care. 2016;54:1105‐1111. 27116111 10.1097/MLR.0000000000000550

[40] Jha AK , Kuperman GJ , Teich JM , et al. Identifying adverse drug events: development of a computer‐based monitor and comparison with chart review and stimulated voluntary report. J Am Med Inform Assoc. 1998;5:305‐314. 9609500 10.1136/jamia.1998.0050305 PMC61304

[41] Classen DC , Pestotnik SL , Evans RS , Burke JP . Computerized surveillance of adverse drug events in hospital patients. JAMA. 1991;266:2847‐2851. 1942452

[42] Musy SN , Ausserhofer D , Schwendimann R , et al. Trigger tool‐based automated adverse event detection in electronic health records: systematic review. J Med Internet Res. 2018;20:e198. 29848467 10.2196/jmir.9901 PMC6000482

[43] Bhise V , Sittig DF , Vaghani V , Wei L , Baldwin J , Singh H . An electronic trigger based on care escalation to identify preventable adverse events in hospitalised patients. BMJ Qual Saf. 2018;27:241‐246. 10.1136/bmjqs-2017-006975 PMC5867429 28935832

[44] Samner C , Miller S , Jones C , et al. Developing and evaluating an automated all‐cause harm trigger system. Jt Comm J Qual Saf. 2017;43:155‐165. 10.1016/j.jcjq.2017.01.004 28325203

[45] National Quality Forum . Cancer 2015–17: Technical report. Washington, DC: National Quality Forum, 2017 Available at: http://www.qualityforum.org/Publications/2017/01/Cancer_2015-2017_Technical_Report.aspx. Accessed August 18, 2019.

